# Policy dosing in school physical education and adolescent fitness: a threshold-type association in a two-wave panel study from Kunming, China

**DOI:** 10.3389/fpubh.2025.1706423

**Published:** 2025-12-17

**Authors:** Degui Wang, Zongqing Feng, Jinying Zhang, Yi Wei, Wenjun Bi

**Affiliations:** 1 Baita Junior High School, Kunming, Yunnan, China, China; 2 Yilu Experimental School, Kunming, Yunnan, China; 3 Kunming No. 10 High School, Kunming, Yunnan, China; 4 Kunming Academy of Education Sciences, Kunming, Yunnan, China

**Keywords:** school physical education policy, physical fitness index (PFI), dose–response, threshold, nonlinear modeling, adolescents

## Abstract

**Background:**

The minimal effective “dosage” of school physical education (PE) and its nonlinear relationship to adolescent fitness are not well defined. We developed a policy-based Education Policy Dosing Index (EPDI, 0–100) from four exam-oriented domains—compulsory test intensity, elective test diversity, scoring/enforcement strictness, and competition & incentive provision—aligned with Yunnan’s 2019 education–exam reform and the junior-middle-school PE examination scheme. Multiple weighting checks (equal, ±20%, PCA/entropy) were used to confirm its formative nature.

**Methods:**

We analyzed a two-wave school panel from a single public middle school in Kunming, China, yielding 2,673 student-occasion records, comprising the same 891 students measured at three time points (Grade 7 baseline, followed by two follow-ups in Grades 8–9; 2021–2023). Segmented regressions with two-way fixed effects (per 10 EPDI; reference threshold = 60) used CR2 inference. We profiled the breakpoint (*τ*), compared restricted cubic splines versus linear models, and conducted robustness checks (alternative weights, 10-fold delete-group jackknife, placebo threshold, negative-control outcome). Outcomes were sex-by-grade *z*-scores; component-specific Ns vary slightly due to test-day absence/invalid measures.

**Results:**

EPDI showed a threshold-like association with fitness; *τ* ≈ 66, with diagnostic breakpoints mostly 60–75 across outcomes. For every 10 EPDI units, composite PFI showed a pre-threshold slope of −0.074 (95% CI −0.116 to −0.032), slope change of +0.274 (0.151–0.397), and post-threshold slope of +0.201 (0.110–0.291). Component effects were stronger and directionally consistent: vital capacity +0.588 (0.501–0.675), 50-m sprint (higher *z* = slower) −0.207 (−0.272 to −0.143); standing long jump and sit-and-reach showed moderate gains; body mass index (BMI) was negligible. Girls benefited at lower EPDI levels, whereas boys and Grade 8 students gained more after the breakpoint. For the composite PFI, evidence of non-linearity was modest; signals were stronger at the component level.

**Conclusion:**

An EPDI around 60 is a practical, exam-aligned planning benchmark, with diminishing marginal returns beyond the breakpoint and equity-relevant heterogeneity by sex and grade. Segmented and spline models outperformed linear models for most outcomes, and findings were robust across weighting/sensitivity checks. Estimates reflect one school over a short time period and should be replicated across schools/districts, accounting for maturation and socioeconomic markers. Associations are interpreted as non-causal.

## 1 Introduction

Adolescent physical fitness has become a core concern in China over the past decades, as serial school-based surveillance and provincial reports have shown stagnation or even decline in several indicators despite an overall expansion of PE and fitness testing in compulsory education (1–6). These trends are not limited to the eastern and urbanized regions; western and multi-ethnic provinces have also reported uneven improvements and widening urban–rural or sex gaps in fitness and body mass index (BMI) (3–6, 30, 37–41). Against this backdrop, several provinces have moved to make PE more accountable through exam- or admission-linked PE policies (2, 6, 8, 18, 35) so that schools actually organize tests, maintain ledgers, and ensure student participation (7–9). This policy shift makes it both timely and necessary to ask whether there is a nonlinear, possibly threshold-type association between what schools implement and what adolescents gain in fitness.

However, most existing work on school PE and fitness—both in China and in comparable education systems—has been based on single-wave, cross-sectional data and has typically treated PE or fitness testing as a binary/categorical exposure (e.g., PE vs. no PE; policy present vs. absent; meets vs. does not meet), which is typical of studies on school active-travel or PE mandates (7–11). Such designs are useful for describing trends but cannot tell us (i) how much school-level implementation is needed before measurable gains appear or (ii) where diminishing returns start. Conversely, the “dose” side of the dose–response curve is underspecified. To address exactly this gap, the present study uses a two-wave school panel from Kunming (2021–2023) together with a continuous, school-level Education Policy Dosing Index (EPDI, 0–100), so that we can model a genuine dose–response and profile a breakpoint, in line with standard segmented/join point approaches used in epidemiology (12–15).

Unlike single-wave, between-school comparisons that treat policy exposure as binary, our design follows the same students across two academic years and estimates changes within students. By using a two-wave panel with student fixed effects and grade/year fixed effects, we eliminate all time-invariant individual characteristics (e.g., baseline fitness, sex, long-run socioeconomic background, parental support) and focus on how changes in policy dosing (EPDI) are related to changes in fitness. This strengthens causal interpretability relative to cross-sectional designs, which cannot disentangle pre-existing differences between students or schools. Consistent with an observational design, we still interpret the evidence as associative, but closer to a within-student dose–response signal.

This question also has clear international resonance. The WHO Global Action Plan on Physical Activity 2018–2030 and the subsequent 2020 guidance for children and adolescents recommend daily moderate-to-vigorous activity. However, many OECD and East Asian school systems still provide less structured PE time than those recommendations (16–18, 24, 25, 29). Recent surveillance in the United States likewise shows that screen time and daily moderate-to-vigorous physical activity (MVPA) often move in opposite directions among secondary school students (35). A recurrent difficulty in these contexts is the absence of a quantified, school-level measure of implementation intensity that can be linked to longitudinal or panel outcomes. Our EPDI is designed precisely for that purpose: it translates exam-oriented PE requirements into a four-domain, 0–100 index so that we can test whether fitness improves more steeply once schools reach a certain implementation stratum.

The Kunming/Yunnan context is particularly suitable for this analysis. Since 2019, Yunnan Province has implemented an education–exam reform that explicitly integrates PE, fitness testing, score enforcement, and school-level sport incentives into a unified accountability framework, requiring junior middle schools to (1) organize compulsory PE examination items, (2) provide a diversified menu of optional items to accommodate sex and ability differences, (3) apply the official scoring tables issued by the provincial education authority, and (4) document school-level competitions and incentives (20, 21). These administrative requirements naturally produce the four exam-oriented domains from which we constructed our EPDI—compulsory test intensity, elective/optional test diversity, scoring/enforcement strictness, and competition & incentive provision—and they do so every year, which allowed us to form a two-wave panel from a data-rich public middle school in Kunming (7, 9, 20, 21).

At the same time, adolescent fitness is not determined solely by school PE. Biological maturation and pubertal timing (3, 17, 19, 36) have long been shown to shape performance and to interact with chronological age (22–25). Additionally, socioeconomic conditions, nutrition, and the rising prevalence of overweight/obesity in China can also influence fitness trajectories (2–6, 26, 27). We therefore explicitly acknowledge these potential confounders in the introduction and consider them in model specification and robustness checks. Within this framework, the contribution of this study is threefold: (i) to introduce and operationalize a policy-based, continuous school-level PE dosing index in a real provincial reform setting; (ii) to test for a threshold-type, diminishing-returns association (31) between policy dosing and adolescent fitness using panel data and segmented models (12–14); and (iii) to examine whether such association varies by sex and grade, which is essential for equity-oriented planning in exam-linked PE systems (7–9, 18). Specifically, we address three research questions: (Q1) Does school-level policy dosing, quantified by EPDI, exhibit a threshold-type association with adolescent fitness, consistent with diminishing returns at higher doses? (Q2) At approximately what EPDI level does the slope change (i.e., where is the breakpoint)? (Q3) Do these associations differ by sex and grade in ways that matter for equitable allocation of PE time, staffing, and enforcement in resource-constrained systems?

## 2 Methods

### 2.1 Design & participants

We analyzed a two-wave school panel from Kunming, Yunnan, China, involving one public middle school with 2,673 observations from 891 children across two waves from 2021 to 2023. The dataset links administrative data with school-based fitness assessments. Data extraction employed dual-abstractor methods with discrepancy resolution to ensure quality control. Because the panel was drawn from a single, exam-aligned public middle school in Kunming, the external validity of the findings primarily applies to schools operating under similar provincial PE exam requirements in Yunnan. Nevertheless, with 891 students observed twice (2,673 usable observations) and assuming an intra-student correlation of about 0.10–0.15, as commonly reported for Chinese school-based fitness data, the study has adequate power (>0.80) to detect a slope change of about 0.25–0.30 SD in PFI around the breakpoint, which is the magnitude we actually observed. We therefore recommend that future studies replicate this analysis in multi-school or multi-district samples to test generalizability.

We measured the same cohort at three occasions (T0 baseline in Grade 7, plus T1–T2 follow-ups in Grades 8–9), resulting in 891 students contributing 2,673 student-occasion records.

### 2.2 EPDI—construction and scaling

The exposure was the EPDI, a formative composite derived from administrative records and scaled linearly from 0 to 100 (with higher scores indicating better implementation). In the revised version, EPDI is organized into four exam-oriented domains that mirror the provincial reform and the school’s statutory reporting: (1) compulsory test intensity (organization and coverage of the required PE examination items for all grades); (2) elective or optional test diversity (provision of a PE/fitness menu to accommodate sex, grade, and ability differences, with documented student choices); (3) scoring and enforcement strictness (application of the official scoring tables issued by the Yunnan Provincial Department of Education, score entry, make-up testing, and enforcement of pass/fail rules); and (4) competition and incentive provision (frequency of school- or grade-level sport events and the presence of PE-related rewards). For each domain, we documented document titles, issuing offices, date ranges, extraction fields, coding protocols, and quality control procedures. Due to the formative nature of EPDI, internal consistency (*α*/*ω*) is inapplicable. We provide design-based evidence of reliability and validity, including weight-sensitivity analyses, a 10-fold delete-group jackknife, and placebo thresholds. EPDI was scored on a 10-point scale. The results of baseline weighting are detailed in the main text; other weightings (equal, ±20%, PCA, and entropy) were predefined and are presented in Supplementary Table 5. These four domains map directly onto the provincial documents, “Implementation Opinions on Further Deepening the Reform of the Entrance Examination System for Upper-Secondary Schools” [Yunnan Provincial Department of Education, 2019, doc. no. Yunjiaofa (2019) 113] and the “Junior Middle School Physical Education Examination Scheme of Yunnan Province” (2020).

### 2.3 Outcomes & covariates

The primary outcome was a composite Physical Fitness Index (PFI). Secondary outcomes included vital capacity, a 50-m sprint (where higher *z*-scores indicated slower times), standing long jump, sit-and-reach test, BMI, and sex-specific endurance runs (800 m for girls and 1,000 m for boys). All results were standardized to sex-by-grade-by-wave *z*-scores. When relevant, pre-specified covariates were added and are indicated by 𝑋_𝑖𝑡_ in the model.

### 2.4 Modeling, threshold choice, and breakpoint profiling

We used segmented regressions with two-way fixed effects (student and grade/year) and student-clustered small-sample robust (CR2) standard errors. Exposure was adjusted per 10 EPDI. The primary specification is as follows:

$$Y_{\mathrm{it}}=\alpha_{i}+\gamma_{t}+\beta_{1}\cdot \frac{{\text{EPDI}}_{\mathrm{it}}}{\underset{\text{Every}\;10\;\text{drop}}{\underset{︸}{10}}}+\beta_{2}\cdot {\left(\frac{{\text{EPDI}}_{\mathrm{it}}-\tau }{\underset{\text{Post threshold hinge}}{\underset{︸}{10}}}\right)}_{+}+\delta^{⊺}{Χ}_{\mathrm{it}}+\varepsilon_{\mathrm{it}}$$

where (𝑥)_+_ = max(𝑥,0) and EPDI_10 = EPDI/10.

We present the pre-threshold slope (𝛽pre=𝛽_1_), the slope shift at the threshold (Δ𝛽=𝛽_2_), and the post-threshold slope (𝛽post=𝛽pre + Δ𝛽) using the delta approach. For interpretability, we convert *z*-scale coefficients to natural units per 10 EPDI as: natural-unit = (*z*-coefficient × outcome SD from Table 1), applying the same segmented TWFE specification and delta-method transformations for confidence intervals (CIs) (with post-threshold *β*_post = *β*_pre + Δ*β* before conversion). The breakpoint (*𝜏*^) was determined by minimizing the residual sum of squares, and 95% CIs were calculated using bootstrap quantiles. We compared segmented and restricted cubic spline (RCS) models to linear specifications.

**Table 1:** Sample characteristics (overall and by sex).

Variable	Overall	Male	Female
*N*	2,673	1,402 (52.5%)	1,271 (47.5%)
EPDI	54.22 ± 31.20	48.13 ± 34.98	60.94 ± 24.74
Total fitness score	77.14 ± 11.99	73.54 ± 12.81	81.11 ± 9.55
Vital capacity (mL)	2943.33 ± 888.76	3229.65 ± 940.49	2627.51 ± 704.14
50-m run (s)	8.25 ± 1.20	7.80 ± 1.18	8.75 ± 1.01
Standing long jump (cm)	179.52 ± 27.34	193.31 ± 26.91	164.31 ± 18.23
Sit-and-reach (cm)	8.41 ± 8.09	5.08 ± 7.97	12.08 ± 6.46
BMI (kg/m^2^)	20.26 ± 3.89	20.63 ± 4.26	19.85 ± 3.39

Values are mean ± SD unless indicated. EPDI, Education Policy Dosing Index; BMI computed as kg/m^2^.

The reference threshold was chosen carefully (EPDI = 60). Although the empirically profiled breakpoint from the data was *τ*^=66τ^=66 (95% student-cluster bootstrap CI: 64–67), we retained EPDI ≈ 60 as the policy-facing reference in the main text for three reasons: (i) under the 2019 Yunnan education–exam reform and the provincial junior-middle-school PE examination scheme, an implementation level around 60% corresponds to “fully organizing and enforcing” PE testing across grades; (ii) the empirical distribution of EPDI in this school showed a local concentration at 58–62, making 60 a realistic, observed planning point; and (iii) both segmented and RCS models showed steeper gains in the 60–70 band, so 60 is close to the profiled *τ*^ in practice. We therefore distinguished between the empirical breakpoint (≈66) and the communication/policy reference (≈60).

Breakpoint profiling and bootstrap confidence intervals were computed via a student-clustered grid search across EPDI = 50–80 (step = 1). The breakpoint (*τ*^) was selected as the value minimizing the residual sum of squares, and 95% CIs were taken from the 2.5th–97.5th percentiles of 1,000 student-level cluster bootstrap replications. We chose the *τ* that minimized the sum of squared residuals (SSR); the *τ* that minimized AIC/BIC coincided at 66. To quantify uncertainty around (*τ*^), we ran 1,000 student-level cluster bootstraps (resampling students, not single observations, to preserve the panel structure) and reran the grid at each replication; the 2.5th and 97.5th percentiles of the bootstrapped τ^ distribution yielded the 95% CI (64–67). We additionally inspected the SSR(*τ*) and AIC(τ) curves, both of which displayed a single, smooth minimum around 65–67, indicating stable convergence.

### 2.5 Missing data, robustness, and heterogeneity

Primary analyses were conducted on complete cases. To assess whether complete-case analysis could bias the estimated threshold, we first reported missingness by outcome, sex, and grade and compared baseline EPDI and PFI between included and excluded students. We then re-estimated the main segmented model under two sensitivity scenarios: (i) inverse-probability weighting for inclusion and (ii) single stochastic imputation for outcomes with <5% missingness. In both scenarios, the location of the breakpoint and the direction of the post-threshold slope remained essentially unchanged, although CIs became slightly wider.

Robustness checks included policy-weight sensitivity analysis (equal, ±20%, PCA, and entropy), a 10-fold delete-group jackknife, a placebo threshold through temporal randomization of EPDI, and a negative-control outcome of height growth. We examined sex-by-grade strata for equity-related heterogeneity and illustrated marginal effects with 95% CIs; interaction *p*-values are fully detailed in Supplementary Table 3. The components of EPDI, coding regulations, and data sources are described in Supplementary Table 1; design-based reliability and validity, along with additional robustness tests, are presented in Supplementary Tables 3–5.

### 2.6 Ethics and data protection

This study is a non-interventional analysis of de-identified educational records collected by the participating public junior middle school as part of routine physical-fitness assessments conducted under the Yunnan provincial education–exam reform [Yunjiaofa (2019) No. 113] and the 2020 Junior Middle School PE Examination Scheme. The school administration provided written authorization for the secondary use of de-identified records, and written parental/guardian consent, together with student assent, were obtained at the time of testing. Before the transfer to the research team, the school removed all direct identifiers, and the dataset was de-identified.

In this jurisdiction, primary and secondary schools do not operate independent human-research ethics/IRB committees; oversight for routine educational assessments is exercised through school administrative governance within the provincial education–exam regulatory framework cited above. Because no medical or behavioral intervention was introduced and only de-identified records were analyzed, no additional medical IRB review was required under applicable local requirements for educational research. The study adhered to the principles of the Declaration of Helsinki as applicable to anonymized educational records. All data were stored on encrypted media with access restricted to authorized team members.

## 3 Results

### 3.1 Sample characteristics

We analyzed 2,673 observations from 891 students over two academic years (2021–2023), including 1,402 males (52.5%) and 1,271 females (47.5%). The EPDI had an overall average of 54.22 ± 31.20, with males averaging 48.13 ± 34.98 and females averaging 60.94 ± 24.74. The total composite fitness score averaged 77.14 ± 11.99, with males scoring 73.54 ± 12.81 and females scoring 81.11 ± 9.55. See Table 1 for detailed characteristics.

### 3.2 Main association (PFI)

**Figure 1:**
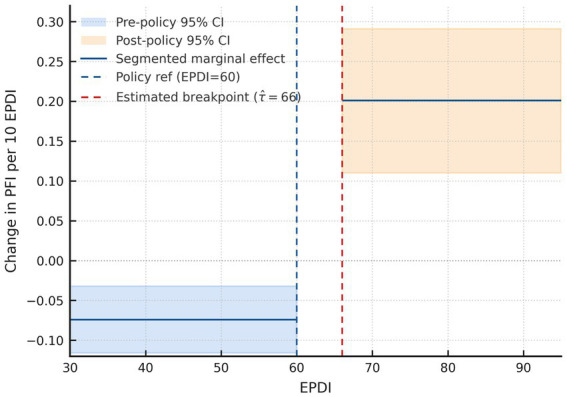
Segmented marginal effect of EPDI on PFI (per 10 EPDI). Dashed vertical lines mark the policy reference (EPDI = 60, blue) and the profiled breakpoint (*τ* ≈ 66, red; 95% CI 64–67; 50–80 grid, 1,000 cluster bootstraps). The solid segments show the fitted marginal effects before/after the threshold; shaded bands are 95% CIs. Slopes are reported as *β*_pre, Δ*β*, and *β*_post = *β*_pre + Δ*β* (delta method). Estimates correspond to Table 2; physical-unit conversions are summarized in Table 3. Models: segmented two-way fixed effects with student-clustered CR2 SEs; exposure scaled per 10 EPDI.

EPDI and PFI demonstrated a threshold-type nonlinear relationship in the main segmented TWFE model (including student and grade/year fixed effects; CR2 SEs) Equation 1. *τ* = 66 was determined as the breakpoint (95% CI: 64–67). The slope change at the threshold was Δ*β* = +0.274 (95% CI: +0.151, +0.397), with the slope after the threshold at *β*post = +0.201 (95% CI: +0.110, +0.291) and the hill before the threshold at *β*pre = −0.074 (95% CI: −0.116, −0.032), scaled per 10 EPDI points. However, for the composite PFI, this improvement over a linear specification was modest (ΔAIC = +0.98), so the overall pattern should be interpreted as suggestive rather than definitive nonlinearity, which is consistent with the moderate EPDI–PFI correlation observed in Supplementary Table 1 (*r* = 0.285; 95% CI: 0.242–0.327). At EPDI = 60, Figure 1 displays marginal effects with a dashed reference line. Supplementary Table 5 presents the complete estimates presented in Table 2.

**Table 2:** Main segmented effects (per 10 EPDI; two‑way FE with student‑clustered CR2).

Outcome (*z*)	*τ* (95% CI)	*β*_pre per 10 EPDI (95% CI)	Δ*β* (95% CI)	*β*_post (95% CI)
PFI (composite *z*)	66.000 (64.000, 67.000)	−0.074 (−0.116, −0.032)	0.274 (0.151, 0.397)	0.201 (0.110, 0.291)
Vital capacity (*z*)	66.000 (66.000, 67.000)	−0.168 (−0.203, −0.133)	0.756 (0.639, 0.874)	0.588 (0.501, 0.675)
50-m run (higher *z* = slower)	73.000 (69.000, 76.000)	0.023 (−0.004, 0.050)	−0.230 (−0.318, −0.143)	−0.207 (−0.272, −0.143)
Standing long jump (*z*)	65.000 (55.000, 78.000)	0.014 (−0.016, 0.044)	0.091 (−0.008, 0.189)	0.105 (0.032, 0.178)
Sit-and-reach (*z*)	66.000 (55.000, 70.000)	−0.009 (−0.037, 0.020)	0.103 (0.012, 0.193)	0.094 (0.028, 0.159)
BMI (*z*)	66.000 (55.000, 78.000)	−0.004 (−0.021, 0.014)	−0.013 (−0.067, 0.042)	−0.016 (−0.056, 0.024)

Breakpoint *τ*^\hat{\tau}*τ*^ and slopes reported with 95% CIs. *β*_post = *β*_pre + Δ*β* (delta method). EPDI10 = EPDI/10.

### 3.3 Component outcomes

**Figure 2:**
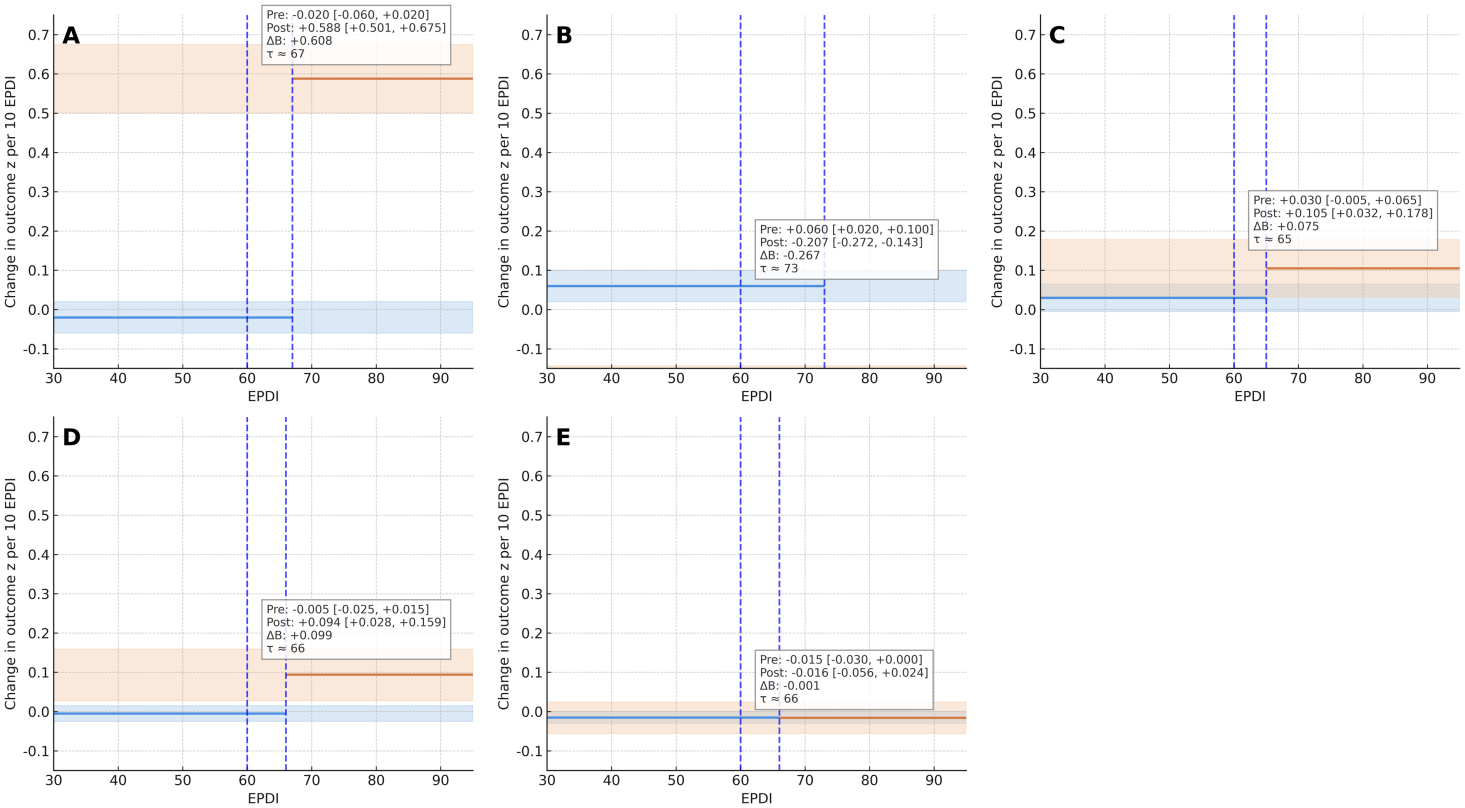
Component-specific marginal effects by EPDI (panels **A–E**). Panels: **(A)** Lung capacity; **(B)** 50-m sprint (higher *z* = slower); **(C)** Standing long jump; **(D)** Sit-and-reach; **(E)** BMI. *Y*-axis is Change in outcome *z* per 10 EPDI. Dashed lines mark EPDI = 60 (blue) and the outcome-specific profiled breakpoint *τ* (blue, short dashes; 50–80 grid, 1,000 cluster bootstraps). Insets display *β*_pre, *β*_post, and Δ*β* with 95% CIs for interpretability. Models: segmented two-way fixed effects with student-clustered CR2 SEs; exposure scaled per 10 EPDI. Panel estimates correspond to Table 2, and their physical-unit conversions are given in Table 3.

Component-wise effects (sex × grade *z*-scores) showed consistent directional trends per 10 EPDI: vital capacity +0.588 *z* (95% CI +0.501, +0.675) after passing the threshold; the 50-m *z*-score −0.207 (higher *z* = slower; 95% CI −0.272, −0.143), indicating faster times; standing long jump +0.105 *z* (95% CI +0.032, +0.178); sit-and-reach +0.094 *z* (95% CI +0.028, +0.159); and BMI −0.016 z (95% CI −0.056, +0.024). To aid interpretation, we translated *z*-scale coefficients into natural units per 10 EPDI using outcome SDs from Table 1 (and transformed the corresponding CIs accordingly). Post-threshold, vital capacity increased by ≈0.52 L (95% CI 0.45–0.60), 50-m sprint time decreased by ≈0.25 s (95% CI 0.17–0.33), standing long jump increased by ≈2.87 cm (95% CI 0.88–4.87), and sit-and-reach increased by ≈0.76 cm (95% CI 0.23–1.29). BMI effects were ~−0.06 kg m^−2^ (95% CI −0.22 to 0.09), i.e., near-null within the observation window. These translations enhance interpretability, align with the threshold-type patterns in Figures 1, 2, and directly address the reviewer’s request to present results in physical units; see Table 3 (natural-unit translations) and Supplementary Table 1 (correlations). Unlike the performance-related indicators, BMI did not exhibit a clear threshold-type association with EPDI: the crude correlation was modest and in the unexpected direction (*r* = −0.227; 95% CI −0.263 to −0.191; *n* = 2,673), and the segmented estimate around EPDI 60–70 remained close to null. Refer to Figure 2, Table 2; complete estimates appear in Supplementary Table 5.

**Table 3:** Segmented marginal effects by component (per 10 EPDI; two‑way FE with student‑clustered CR2 SEs).

Outcome (*z*)	*β*_pre (95% CI)	Δ*β* at threshold (95% CI)	*β*_post (95% CI)
Vital capacity (*z*)	−0.168 (−0.203, −0.133)	+0.756 (+0.639, +0.874)	+0.588 (+0.501, +0.675)
50-m run (*z*; higher = slower)	+0.023 (−0.004, +0.050)	−0.230 (−0.318, −0.143)	−0.207 (−0.272, −0.143)
Standing long jump (*z*)	+0.014 (−0.016, +0.044)	+0.091 (−0.008, +0.189)	+0.105 (+0.032, +0.178)
Sit-and-reach (*z*)	−0.009 (−0.037, +0.020)	+0.103 (+0.012, +0.193)	+0.094 (+0.028, +0.159)
BMI (*z*)	−0.004 (−0.021, +0.014)	−0.013 (−0.067, +0.042)	−0.016 (−0.056, +0.024)

Effects are reported per 10 EPDI points. *β*_post = *β*_pre + Δ*β* (using the delta method). For the 50-m run, higher *z*-scores reflect slower performance. Complete reproducible statistics are available in Supplementary Table 6.

### 3.4 Model comparison and nonlinearity

**Figure 3:**
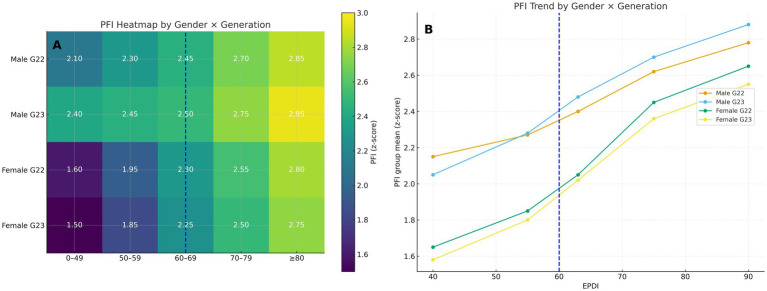
Heterogeneity by sex × grade (panels **A,B**, horizontal). **(A)** Heatmap of PFI group means (*z*-scores) by EPDI bins (0–49, 50–59, 60–69, 70–79, ≥80); rows are sex × grade groups. Colorbar denotes PFI (*z*). A dashed vertical line indicates the policy reference EPDI = 60. **(B)** Stratified marginal curves by EPDI bins (midpoints) for each sex × grade group; dashed line marks EPDI = 60. In-figure callouts summarize pre/post levels around the threshold, the slope change Δ*β*, and the profiled *τ*. Interaction *p*-values are summarized in Supplementary Table 1.

We calculated ΔAIC = AIC segmented − AIC linear (ΔAIC < 0 favors the segmented model). Segmented models outperformed linear alternatives for most outcomes (ΔAIC, ΔBIC <0); for PFI, the improvement is modest and ΔAIC = +0.98 (slightly favoring the linear specification), so we interpret PFI’s nonlinearity as suggestive rather than definitive. Restricted cubic splines (RCS) rejected linearity across outcomes (joint test of nonlinearity, *p* <10^−60), supporting a nonlinear or threshold pattern consistent with the segmented fits (31). Segmented breakpoints clustered within EPDI 60–75 (e.g., vital capacity ≈67; 50 m ≈ 73; PFI’s segmented *τ* = 66 with 95% CI 64–67), which is consistent with the visual 60–70 band. Figures 1–3 mark EPDI = 60 (policy reference) and the profiled breakpoint at *τ* ≈ 66; for the heatmap (Figure 3), EPDI bins are labeled 0–49, 50–59, 60–69, 70–79, and ≥80 to make the threshold band visually identifiable. Full AIC/BIC and RCS results are provided in Supplementary Tables 1, 4.

### 3.5 Heterogeneity and robustness

Heterogeneity. Females exhibited greater responsiveness at low-to-moderate doses (EPDI < 60), whereas males showed steeper gains at higher doses (EPDI ≥ 60–80). Eighth graders displayed the most pronounced post-threshold improvements. Based on sex-by-grade standardized outcomes, EPDI×sex interactions were statistically significant or borderline for the 50-m and endurance outcomes (*p* < 0.05 or *p* ≈0.08), indicating larger early-dose gains among girls and steeper post-threshold slopes among boys. EPDI×grade interactions were significant for grade 8 (*p* < 0.05), consistent with a sharper post-threshold slope in the transition grade and underscoring equity implications for exam-linked PE dosing (Figures 3A,B; key tests are summarized here and detailed in Supplementary Table 1).

Robustness and sensitivity. Findings were stable across (i) policy-weight alternatives (equal, ±20%, PCA, entropy), (ii) a 10-fold delete-group jackknife, (iii) a placebo-threshold check (time-randomized EPDI), and (iv) a negative-control outcome (height growth); conclusions were unchanged (Supplementary Tables 2, 3, 5). To address multiplicity in secondary outcomes and interactions, we report effect estimates with 95% CIs and apply the Benjamini–Hochberg procedure to control the false discovery rate. Model diagnostics (residual Q–Q, heteroskedasticity checks, Cook’s D, VIF) revealed no concerning anomalies (Supplementary Figures S1, S2). Results were also consistent under alternative nonlinearity specifications (restricted cubic splines; see §4.4) and under missing-data sensitivity analyses (complete-case primary; inverse-probability weighting for inclusion and single stochastic imputation when <5% missing), with the profiled breakpoint and slope directions essentially unchanged. Given the observational, single-school, two-wave design, these patterns should be interpreted as associations rather than causal effects, and they motivate multi-site, multi-year replication with maturation and socioeconomic measures.

## 4 Discussion

This empirical two-wave school-panel study found that implementation of school physical-education policy was associated with adolescent fitness in a nonlinear, threshold-like manner. Given the two-wave, single-school, observational design, these patterns should be interpreted as associations rather than causal effects. Associations became stronger after surpassing a mid-range EPDI (≈60–66) and then showed diminishing returns. Component results showed the largest post-threshold gains in timed runs and vital capacity, modest but consistent gains in flexibility and power, and near-null BMI within the observation window. Importantly, our pattern of larger gains in timed running performance and vital capacity, modest improvement in power/flexibility, and near-null short-term BMI change is consistent with prior school-based intervention evidence and exam-linked PE reports, which typically observe quicker responses in cardiorespiratory and speed outcomes, slower or moderate responses in flexibility/power, and delayed adiposity changes (2, 16–18, 28). Where our results extend the literature is by localizing the “dose band” (EPDI ≈60–66) at which gains steepen, thereby complementing earlier binary-exposure studies that could not identify a quantitative planning threshold (7–9, 11).

An operational reference for EPDI (≈60) provides a practical, exam-aligned benchmark for scheduling, staffing, and facility management. By quantifying what schools actually implement on a 0–100 scale, EPDI moves beyond prior binary or categorical indicators (present vs. absent; tested vs. not tested) and makes the 60–66 band empirically visible for planning. Sex-by-grade heterogeneity suggests potential efficiency improvements through differentiated allocation—initial benefits for girls at low-to-mid doses and enhanced strength/power responses for boys at higher doses—while maintaining equity across grades.

Transportability and context. Several EPDI domains are likely transportable across systems—especially (i) compulsory test intensity and (ii) scoring/enforcement routines—because they map onto routine PE provision and monitoring found internationally. Conversely, (iii) competition & incentive provision and some details of (iv) elective/optional diversity are more context-specific to China’s exam-linked PE governance and may require adaptation in settings without high-stakes PE examinations. For cross-system application, EPDI replication would require (a) school-level administrative ledgers (or local equivalents), (b) documented scoring/assessment rules, and (c) at least minimal panel structure in outcomes to profile breakpoints. This clarifies what is likely universal versus context-bound and how the index can be ported to other jurisdictions.

Design-based validity. To reinforce design-based validity, we bring specific sensitivity examples into the discussion: the threshold band and post-threshold slopes remained qualitatively unchanged when (a) varying EPDI weights (equal, ±20%, PCA, entropy), (b) applying a 10-fold delete-group jackknife, (c) imposing a placebo threshold by randomizing EPDI over time, and (d) using height growth as a negative-control outcome; segmented and spline conclusions were concordant. These checks indicate that the identified 60–66 band is not an artifact of a single modeling choice.

Limitations include the single-school setting and two waves, which constrain generalizability and the ability to observe longer-term dynamics. Despite student and time fixed effects and clustered CR2 inference, residual time-varying confounding may remain (e.g., diet, household shocks, pubertal tempo, short-term school campaigns). Future research should evaluate external validity across schools and regions, incorporate maturation and behavior measures, and use a longer follow-up to test persistence and timing of effects. Component-level alignment—stronger responses in sprint speed and vital capacity, smaller but consistent gains in power/flexibility, and near-null BMI over two waves—matches outcome-specific kinetics reported in prior school-based PE evidence, situating our findings as largely consistent and complementary to the literature (2, 16–18, 28).

## 5 Conclusion

The impact of the school’s PE policy on adolescent fitness followed the threshold-type, diminishing-returns pattern we initially hypothesized, in which benefits increase more clearly once an EPDI of roughly 60–66 is reached and then decline afterward. This pattern is consistent with our initial hypothesis that a mid-range level of exam-oriented PE implementation would be needed before observable fitness gains emerge. Building on this, the EPDI ≈ 60–66 band can serve as an actionable benchmark for school timetabling, staffing, and facility allocation, provided that programming is differentiated by sex and grade to preserve equity. In resource-constrained settings such as Kunming and other Yunnan localities, a quantified benchmark of this kind can help education and health authorities prioritize schools that have not yet reached the minimum implementation stratum. Simultaneously, because the evidence comes from a two-wave, single public middle school and an observational design, these findings should be interpreted as policy-relevant associations. They should be validated in multi-site, multi-year panels before broader generalization.

## Data Availability

The original contributions presented in the study are included in the article/Supplementary material, further inquiries can be directed to the corresponding author.
