# Supplementary material for: Policy dosing in school physical education and adolescent fitness: a threshold-type association in a two-wave panel study from Kunming, China
Source: Front Public Health. 2025 Dec 17;13:1706423. doi: 10.3389/fpubh.2025.1706423 (PMC12753875; doi:10.3389/fpubh.2025.1706423)
Supplement: Supplementary file 1 [file Table_1.docx]

## Table S1 Correlations between EPDI and fitness outcomes (Pearson r, 95% CI)

| Outcome | n | r | 95% CI (lower) | 95% CI (upper) | Direction as expected? |
| --- | --- | --- | --- | --- | --- |
| 1000-m run | 1402 | -0.712 | -0.737 | -0.685 | Yes |
| 50-m sprint (higher z = slower) | 2673 | -0.346 | -0.379 | -0.312 | Yes |
| 800-m run | 1271 | -0.749 | -0.772 | -0.724 | Yes |
| BMI | 2673 | -0.227 | -0.263 | -0.191 | No |
| PFI (composite) | 1782 | 0.285 | 0.242 | 0.327 | Yes |
| 1-min sit-ups | 1271 | 0.254 | 0.201 | 0.304 | Yes |
| Sit-and-reach | 2673 | 0.325 | 0.291 | 0.359 | Yes |
| Pull-ups | 1402 | 0.372 | 0.326 | 0.416 | Yes |
| Standing long jump | 2673 | 0.273 | 0.238 | 0.308 | Yes |
| Vital capacity | 2673 | 0.177 | 0.140 | 0.213 | Yes |

Note: This table follows a three-line-table structure (top rule, header rule, bottom rule) as commonly used in journals. Time-based items (50 m, 800 m, 1000 m) were coded as “higher z = slower”, so the expected association with EPDI is negative. 95% CI was calculated using Fisher-z transformation.
