# Supplementary material for: Policy dosing in school physical education and adolescent fitness: a threshold-type association in a two-wave panel study from Kunming, China
Source: Front Public Health. 2025 Dec 17;13:1706423. doi: 10.3389/fpubh.2025.1706423 (PMC12753875; doi:10.3389/fpubh.2025.1706423)
Supplement: Supplementary file 2 [file Table_2.docx]

Table S2. Model diagnostics: segmented vs. linear (AIC/BIC and fit statistics)

| Outcome | Empirical_breakpoint | AIC_linear | AIC_segmented | Delta_AIC(seg-lin) | BIC_linear | BIC_segmented | Delta_BIC(seg-lin) | N |
| --- | --- | --- | --- | --- | --- | --- | --- | --- |
| LungCapacity | 67.0 | 36216.627 | 35267.366 | -949.261 | 36228.409 | 35285.038 | -943.37 | 2673 |
| LongJump | 72.0 | 17482.378 | 16751.139 | -731.239 | 17494.16 | 16768.812 | -725.348 | 2673 |
| SitReach | 74.0 | 10878.488 | 10818.76 | -59.728 | 10890.27 | 10836.432 | -53.837 | 2673 |
| Sprint50m | 73.0 | 646.936 | -30.205 | -677.141 | 658.718 | -12.532 | -671.25 | 2673 |
| BMI | 68.0 | 7124.035 | 7063.746 | -60.289 | 7135.817 | 7081.419 | -54.398 | 2673 |
| PFI | 74.0 | -931.331 | -930.349 | 0.981 | -920.36 | -913.893 | 6.467 | 1782 |

Notes: Negative ΔAIC/ΔBIC indicates preference for segmented over linear; lower is better. All models estimated under two-way fixed effects; CR2 SEs; exposure per 10 EPDI.
**Table 2. Main segmented effects (per 10 EPDI; two‑way FE with student‑clustered CR2)**

| Outcome (z) | τ̂ (95% CI) | β_pre per 10 EPDI (95% CI) | Δβ (95% CI) | β_post (95% CI) |
| --- | --- | --- | --- | --- |
| PFI (composite z) | 66.000 (64.000, 67.000) | -0.074 (-0.116, -0.032) | 0.274 (0.151, 0.397) | 0.201 (0.110, 0.291) |
| Vital capacity (z) | 66.000 (66.000, 67.000) | -0.168 (-0.203, -0.133) | 0.756 (0.639, 0.874) | 0.588 (0.501, 0.675) |
| 50‑m run (higher z = slower) | 73.000 (69.000, 76.000) | 0.023 (-0.004, 0.050) | -0.230 (-0.318, -0.143) | -0.207 (-0.272, -0.143) |
| Standing long jump (z) | 65.000 (55.000, 78.000) | 0.014 (-0.016, 0.044) | 0.091 (-0.008, 0.189) | 0.105 (0.032, 0.178) |
| Sit‑and‑reach (z) | 66.000 (55.000, 70.000) | -0.009 (-0.037, 0.020) | 0.103 (0.012, 0.193) | 0.094 (0.028, 0.159) |
| BMI (z) | 66.000 (55.000, 78.000) | -0.004 (-0.021, 0.014) | -0.013 (-0.067, 0.042) | -0.016 (-0.056, 0.024) |

**Notes:** Effects are expressed per 10 EPDI. β_post = β_pre + Δβ (delta method). 50‑m run uses z‑scores where higher values indicate slower performance. Full reproducible statistics are provided in Supplementary Table S5.
