# Supplementary material for: Policy dosing in school physical education and adolescent fitness: a threshold-type association in a two-wave panel study from Kunming, China
Source: Front Public Health. 2025 Dec 17;13:1706423. doi: 10.3389/fpubh.2025.1706423 (PMC12753875; doi:10.3389/fpubh.2025.1706423)
Supplement: Supplementary file 3 [file Table_3.docx]

*Table S3 Delete-group jackknife results for segmented model*

| fold | n | tau_hat | beta_pre | delta | beta_post |
| --- | --- | --- | --- | --- | --- |
| 1 | 1602 | 10.725 | 0.172 | -0.006 | 0.166 |
| 2 | 1604 | 10.725 | 0.167 | -0.005 | 0.162 |
| 3 | 1604 | 10.725 | 0.147 | -0.004 | 0.143 |
| 4 | 1604 | 10.725 | 0.177 | -0.005 | 0.172 |
| 5 | 1604 | 10.725 | 0.176 | -0.005 | 0.171 |
| 6 | 1604 | 10.725 | 0.164 | -0.005 | 0.159 |
| 7 | 1604 | 10.725 | 0.170 | -0.006 | 0.164 |
| 8 | 1604 | 10.725 | 0.155 | -0.005 | 0.150 |
| 9 | 1604 | 10.725 | 0.170 | -0.005 | 0.165 |
| 10 | 1604 | 10.725 | 0.163 | -0.005 | 0.158 |
