# Supplementary material for: Policy dosing in school physical education and adolescent fitness: a threshold-type association in a two-wave panel study from Kunming, China
Source: Front Public Health. 2025 Dec 17;13:1706423. doi: 10.3389/fpubh.2025.1706423 (PMC12753875; doi:10.3389/fpubh.2025.1706423)
Supplement: Supplementary file 4 [file Table_4.docx]

Table S4. Restricted cubic spline (RCS) vs. linear: joint nonlinearity tests and fit comparison

| Outcome | Spline_df | AIC_spline | BIC_spline | AIC_linear | BIC_linear | ΔAIC(spline-lin) | ΔBIC(spline-lin) | Nonlinearity_F | Nonlinearity_p |
| --- | --- | --- | --- | --- | --- | --- | --- | --- | --- |
| LungCapacity | 4 | 33914.146 | 33937.71 | 36216.627 | 36228.409 | -2302.481 | -2290.699 | 1034.562 | 0.0 |
| LongJump | 4 | 19088.078 | 19111.642 | 17482.378 | 17494.16 | 1605.7 | 1617.482 | 355.046 | 2.612760540144778e-245 |
| SitReach | 4 | 10857.9 | 10881.464 | 10878.488 | 10890.27 | -20.588 | -8.806 | 91.007 | 1.2794756592711756e-72 |
| Sprint50m | 4 | 4347.438 | 4371.002 | 646.936 | 658.718 | 3700.502 | 3712.284 | 379.031 | 9.922085205346617e-259 |
| BMI | 4 | 8835.875 | 8859.439 | 7124.035 | 7135.817 | 1711.84 | 1723.622 | 81.629 | 1.8976786072697654e-65 |
| PFI | 4 | -453.656 | -431.714 | -931.331 | -920.36 | 477.675 | 488.646 | 679.074 | 0.0 |

Notes: Joint nonlinearity p-values from Wald/F-tests on spline terms; smaller p indicates stronger evidence against linearity. All models include two-way fixed effects and use per 10 EPDI scaling.
