# Supplementary material for: Policy dosing in school physical education and adolescent fitness: a threshold-type association in a two-wave panel study from Kunming, China
Source: Front Public Health. 2025 Dec 17;13:1706423. doi: 10.3389/fpubh.2025.1706423 (PMC12753875; doi:10.3389/fpubh.2025.1706423)
Supplement: Supplementary file 5 [file Table_5.docx]

**Table S5. Robustness under the baseline EPDI weighting (per 10 EPDI; two‑way FE with student‑clustered CR2)**

| Outcome (z) | τ̂ (95% CI) | β_pre (95% CI) | Δβ (95% CI) | β_post (95% CI) | N | Clusters |
| --- | --- | --- | --- | --- | --- | --- |
| PFI (composite z) | 66.000 (64.000, 67.000) | -0.074 (-0.116, -0.032) | 0.274 (0.151, 0.397) | 0.201 (0.110, 0.291) | 1782 | 891 |
| Vital capacity (z) | 66.000 (66.000, 67.000) | -0.168 (-0.203, -0.133) | 0.756 (0.639, 0.874) | 0.588 (0.501, 0.675) | 2673 | 891 |
| 50‑m run (higher z = slower) | 73.000 (69.000, 76.000) | 0.023 (-0.004, 0.050) | -0.230 (-0.318, -0.143) | -0.207 (-0.272, -0.143) | 2673 | 891 |
| Standing long jump (z) | 65.000 (55.000, 78.000) | 0.014 (-0.016, 0.044) | 0.091 (-0.008, 0.189) | 0.105 (0.032, 0.178) | 2673 | 891 |
| Sit‑and‑reach (z) | 66.000 (55.000, 70.000) | -0.009 (-0.037, 0.020) | 0.103 (0.012, 0.193) | 0.094 (0.028, 0.159) | 2673 | 891 |
| BMI (z) | 66.000 (55.000, 78.000) | -0.004 (-0.021, 0.014) | -0.013 (-0.067, 0.042) | -0.016 (-0.056, 0.024) | 2673 | 891 |

**Notes:** Effects are per 10 EPDI. β_post = β_pre + Δβ (delta method). τ̂ 95% CIs via profile‑RSS with bootstrap quantiles. Two‑way fixed effects (student × grade/year) with student‑clustered small‑sample robust errors (CR2). 50‑m run uses z‑scores where higher = slower.
