# Supplementary material for: Policy dosing in school physical education and adolescent fitness: a threshold-type association in a two-wave panel study from Kunming, China
Source: Front Public Health. 2025 Dec 17;13:1706423. doi: 10.3389/fpubh.2025.1706423 (PMC12753875; doi:10.3389/fpubh.2025.1706423)
Supplement: Supplementary file 6 [file Table_6.docx]

Table S6. Natural-unit translations of segmented model effects per 10 EPDI

| **Outcome (unit)** | **Pre-threshold slope (per 10 EPDI)** | **Slope change Δ** | **Post-threshold slope (per 10 EPDI)** |
| --- | --- | --- | --- |
| Vital capacity (mL) | −149 (95% CI −180 to −118) | +672 (+567 to +777) | +523 (+445 to +601) |
| 50 m sprint (s; lower = faster) | +0.03 (−0.01 to +0.06) | −0.28 (−0.38 to −0.17) | −0.25 (−0.33 to −0.17) |
| Standing long jump (cm) | +0.38 (−0.44 to +1.20) | +2.49 (−0.22 to +5.17) | +2.87 (+0.88 to +4.87) |
| Sit-and-reach (cm) | −0.07 (−0.30 to +0.16) | +0.83 (+0.10 to +1.56) | +0.76 (+0.23 to +1.29) |
| BMI (kg/m²) | −0.02 (−0.08 to +0.05) | −0.05 (−0.26 to +0.16) | −0.06 (−0.22 to +0.09) |

Notes: Translated from z-scale using Table 1 SDs (50 m: 1.20 s; vital capacity: 888.76 mL; standing long jump: 27.34 cm; sit-and-reach: 8.09 cm; BMI: 3.89 kg·m⁻²). PFI is a composite z-score and is not expressed in natural units.
